# Recommendations for special challenges faced by technical personnel with geriatric patients in the sleep laboratory – a consensus statement

**DOI:** 10.1007/s11325-025-03482-1

**Published:** 2025-10-21

**Authors:** Nikolaus Netzer, Helmut Frohnhofen, Sven Stieglitz, Esther Marasanov, Andreas Schlesinger, Petra Netzer

**Affiliations:** 1 University Hospitals Div. Sport Medicine, University Ulm, Ulm, Deutschland; 2 Hermann Buhl Institute of Hypoxia and Sleep Medicine Research, Bad Aibling, Germany, Honorary Professorship of the Leopold Franzen’s University, Innsbruck, Austria; 3 Institute of Mountain Emergency Medicine, Eurac Research, Via Hypathia 2, Bolzano, 39100 Italien; 4 Division of Geriatric Traumatology, University Hospitals Düsseldorf, Heinrich Heine University Düsseldorf, Düsseldorf, Germany; 5 Department of Pneumology and Sleep Medicine, Petrus Hospital of the Cellitinnen, Wuppertal, Germany, University of Witten-Herdecke, Faculty of Health, Wuppertal, Germany; 6 Competence Center of Sleep Medicine, Charite University Hospitals, Berlin, Germany; 7 Department of Internal Medicine, St Mary’s Hospital of the Cellitinnen, Cologne, Germany

**Keywords:** Polysomnography, Technical sleep laboratory personnel, Elder, Geriatric persons electrophysiologic signals, Special care

## Abstract

**Background:**

Sleep disorders increase with increasing age. In the USA, 50% of the population 65 yrs and older complain about sleep problems. Therefore, older patients make up a large portion of clients in the sleep laboratory with polysomnography for diagnosis and therapy.

**Methods:**

The technical staff faced with older clients in the sleep laboratory experience several special challenges with this cohort, which are discussed and given recommendations to deal with them in this statement by consensus

**Results and Recommendations:**

Technicians must be aware of different normal values of sleep stage distribution, arousals, sleep efficiency and mean oxygen saturation. The changes in electrophysiologic signals with lower amplitudes in the electroencephalogram of older people increase the difficulties to distinguish between sleep stages. Older adults present with dryer skin which leads to higher electric resistance, weakening electrophysiologic signal quality. The automatic analysis of polysomnography devices can most often not be applied and electrophysiologic channels must be analyzed manually. Older sleep laboratory clients often need more care during the night and home polysomnographies are difficult to perform. Nycturia, the risk of falls and reduced cognitive function with disorientation during the night in elderly increase these difficulties. Special challenges can come from dentures worn or taken out at night, a more often tendency to keep the mouth open during breathing, being frightened to wear a nasal or full-face mask, not understanding the procedure and the need for therapy overall and the forced influence and interventions of relatives before, during and after polysomnography. The collaboration with relatives on the other hand is often needed to perform the diagnostic and therapeutic procedures.

## Introduction

50% of the US American population aged 65yrs and older complain about sleep disorders compared to 15.9–22.3% of the general population [1]. This means that nowadays older adults make up for most clients in sleep laboratories (sleep labs) for polysomnography (PSG) or for home sleep studies. With the demographic development in Western societies the number of older people in the need for diagnostic and therapeutic procedures in sleep medicine will further grow.

Some reasons for a growing number of increasing elderly clients in sleep laboratories beside the demographic development are a higher awareness for sleep disorders associated with menopause and perimenopause in women, a higher awareness for sleep disorders in the elder population, in physicians and caregivers through media and the promising number of studies which have shown that an early onset of therapy in older persons with sleep disordered breathing can save from early loss of cognition or even improve cognition in patients with mild to moderate dementia [2, 3]. However, compared to pediatric sleep medicine training, where physicians and in part technicians receive a specialized training for their clientele since a few decades, the recognition of the fact, that older patients require special knowledge of sleep physicians and especially sleep lab technicians, is just on the rise. There are several special challenges with older sleep patients that sleep lab technicians are facing. These are the most important challenges with older adults based on our experience and as described in the scientific literature:

1. Electrophysiologic signals of older sleep laboratory patients differ in part from younger or middle-aged subjects due to a lower voltage of neuronal electric ouput. The amplitudes of all waves, but especially Delta waves, are lower in older adults. Additionally, REM (Rapid Eye Movement) intensity during REM-phases is also lower. Older adults present with dryer skin which leads to higher electric resistance, weakening electrophysiologic signal quality. Technicians must be aware of the different normal values of sleep stage distribution, arousals, sleep efficiency and mean oxygen saturation. This makes analysis of the sleep parameters and determination of sleep phases more difficult. Automatic analysis programs of electrophysiologic signals in polysomnography (PSG) devices reveal therefore in part wrong results [2, 4–6].
2. Respiratory signals differ in part from younger or middle-aged adults due to changes in lung diffusion, loss of active lung tissue and more restricted lung function. The average basic mean oxygen saturation is therefore lower, breathing frequency in part higher and flow volume curves lower. The loop gain after an apnea or hypopnea is lower in older humans. Central apneas without pathologic background increase with age, as do mixed apneas due to aging and changes in the chemosensitivity of the peripheral chemoreceptors for CO2 (Carbon dioxide). Persons age >75yrs. have often a combination of sleep disordered breathing with COPD (Chronic obstructive pulmonary disease), the so-called overlap syndrome, and need a combination treatment [2, 4–6].
3. The usual screening and pretest probability instruments like the Epworth Sleepiness Scale, the Berlin Questionnaire and the Stop-Bang among others have limited use in older persons with suspected sleep disorders. Technicians in sleep labs, who rely on these pre-informative instruments, might be misled by their information and the older clientele might be confused by some of the questions. For example, many older persons do not drive a car anymore and cannot give answers concerning tiredness behind the wheel in the Berlin Questionnaire, but the common shorter duration of continuous sleep in the first half of the night leads also to a wrong perception of tiredness and false reporting in the Epworth Sleepiness Scale [7–11].
4. Several comorbidities in elder sleep lab patients are age-related rather than caused by sleep disordered breathing, for example high blood pressure, nycturia and others. It is often not easy to distinguish what the cause and what is the effect between the comorbidities (i.e. nycturia) and advise the patient accordingly or give the right diagnosis. Sleep disorders treatment (i.e. PAP device) will likely interfere with the already existing medication (i.e. High blood pressure medication) because the treatment changes the physical preconditions of the patient. This must be considered at an early stage of the sleep laboratory procedure by the sleep laboratory technician [12–14].
5. Due to nycturia and other age-related problems, older patients in the sleep laboratory tend to get out of the bed during a PSG more often than middle-aged patients. This implicates the risk of falls in the sleep laboratory or during an PSG in the home environment. Special care and attention are therefore needed with seniors in the sleep laboratory [11].
6. A cognitive decline with higher age is common in several patients. This implicates longer explanation times concerning the PSG procedure, the need for therapy and the usage of mask and PAP device or certain medications by technical staff. Some patients might not have the cognitive capabilities to cooperate with staff at all and relatives or caregivers must be included in the processes [15–18].
7. Older patients >75 yrs. often have a masseter muscle weakness and tend to keep their mouth open while sleeping. This often makes technical staff in the sleep lab unsecure and leads to immediate use of full-face mask in those patients, which in the long run can then lead to lower compliance as well as medical problems [19–23].
8. A proportion of seniors is edentulous and uses or don’t use dentures at night. This can have several implications: respiratory values can differ between wearing dentures during polysomnography or not. It makes a difference for the mask fitting if dentures are worn during nasal or full-face mask use with PAP devices. With nasal mask there is a higher risk of leakage problem if dentures are not worn during sleep. Treatment of an edentulous patient with a mandibular advancement device or oral appliance might be difficult or not possible as there are no teeth to properly anchor the device [24–27].
9. Digitalized patient history, as planned for example with the electronic patient history file on the insurance chip card of the health insurances in Germany or is already existing in Health Maintenance Organizations (HMOs) in the United States, has advantages, but can also be misleading for the sleep laboratory staff concerning diseases and problems, which might not be affecting the persons situation in the sleep laboratory and during PSG. For example: the electronic patient file might contain a former psychiatric or neurologic diagnosis, which has meanwhile been reversed, but has never been deleted out of the electronic patient file on the chip card. This may also lead to a prejudiced handling of certain patients [28–30].

## Recommendations on consensus for sleep technicians handling geriatric patients in the sleep laboratory

### Methods

Based on the experience of challenges faced by elderly patients in the sleep laboratory, which have been discussed by members of our assemblies at the 2024 annual conference of the German Sleep Society in Essen, Germany as well as written reports of members of the Certified Sleep Technicians of the German Sleep Society via email to assembly chairs, the assembly chairs voted on recommendations. Assembly chairs came to the following recommendations by consensus of countermeasures for challenges in handling geriatric patients. These are recommendations for sleep laboratory staff members, specifically sleep technicians.

### Results and Recommendations

1. Due to the differences between older persons and young and middle-aged adults concerning the strength of electrophysiologic signals and the differences in sleep structure, the analysis of the electrophysiologic parameters EEG (electroencephalogram); EOG (electrooculogram) and EMG (electromyogram) in polysomnograms of persons >70years of age should be done manually rather than with the automated analysis of the PSG device.
2. The assessment of the polysomnogram regarding “normal” values for sleep stages should be done taking into account the differences between younger and middle-aged adults.
3. An automated analysis of the respiratory channels in a PSG of an elder client is possible. The normally lower levels of mean SaO2 should be considered and automated given values of the oxygen desaturation index inspected and controlled. The assessment of the respiratory parameters should take a normal higher number of respiratory events in older people into account and consider it non pathologic.
4. Pretest results of screening instruments or questionnaires should be reviewed critically. If feasible, an oral interview by the sleep lab staff is helpful to better interpret questionnaire results. If possible, questionnaires specifically designed for elderly like the EFAS (Essen Inventory for Assessment of Sleep Disorders in Older Persons), the ODSI (Observation and Interview Based Diurnal Sleepiness Inventory), or the ESS (Epworth Sleepiness Scale) for elderly should be used [28–31].
5. An assessment of the medications taken by the geriatric patient before PSG is not only necessary in the patient’s interview by a sleep physician but must be repeated orally and confirmed by the sleep laboratory staff before the first night with PSG. The patient might have changed medication in between or take additional medication, which he did not report to the physician. Not too rarely older patients take extra sleeping pills to accommodate the unknown environment in the sleep lab. In addition, extra alcohol intake or the opposite trying to stay sober during the PSG occur quite often. The later occurs more often in elderly patients, who want to make a good impression due to their stricter education as a child. Sleep technicians must take that into account when analyzing PSG data.
6. Patients with severe comorbidities, which is more often the case in older patients >75yrs., such as uncontrolled arterial blood pressure, heart failure of a higher degree, instable blood sugar levels and instable psychological status in patients with psychiatric disorders and dementia require extra care during PSG. Sleep physicians and technicians should discuss such cases in advance and determine together if a patient is fit enough for an ambulant PSG or needs a stationary environment with full night shift nursing during the PSG. Sleep technicians must have a chance to express their concerns in such cases.
7. The tendency of older clients in the sleep laboratory to get up and out of bed more often during the night must be considered. The detaching process from the PSG main device or the PAP device must be explained carefully to the patient and the patient should practice with the technician how it is done before the PSG starts. A urine bottle for elderly men should be provided as standard, for elderly women with reduced cognitive function a constant nursing night shift helping her to find the bathroom might be necessary.
8. The recommendations in point 6 and 7 lead to the conclusion and recommendation that at home PSG in geriatric patients should only be performed if relatives or caregivers, who are informed about and trained in the PSG procedure as well as the process of detaching and put back on the device, are on site during the whole night and willing to provide support. In lab PSG should be the primary choice for elderly and very old clientele.
9. In cases of advanced dementia extra time must be planned by the sleep laboratory staff for talking and explaining to the patient as well as extra care during the PSG (putting electrodes or the mask back on in lost and maybe panicking patients diagnosed with dementia). This can require reducing the numbers of clients in the sleep lab during PSG nights with cognitively impaired patients. The special needs and efforts of technicians during such night must be considered. Sleep physicians must give clear instructions to sleep lab staff after the patient interview for the handling of cognitively impaired patients [14]. These instructions can include to ask relatives for support during the PSG and in some cases have relatives or caregivers sleep in the same room as the patient.
10. First therapy nights with PAP in older and very old patients should always be performed with a nasal mask. If the leakage at higher pressure levels in patients with open mouth is still too high, a therapy trial with a chin strap should be the next step. A full-face mask should be the last step if everything else failed.
11. Edentulous patients who have dentures should primarily be asked if they wear their dentures at night. If feasible, two diagnostic PSG’s, one with and one without dentures are recommended to see differences. In case there are no differences, a PAP trial without dentures with nasal mask should be undertaken. If mask seals without dentures no further steps are needed. If nasal mask doesn’t seal a second PAP trial with dentures should be undertaken and the patient be advised to wear the dentures when sleeping with a PAP nasal mask. In this case the patient should be instructed for special cleaning processes of the dentures and the gum before and after the night’s sleep.
12. Sleep lab staff, physicians and technicians, should be critical towards diagnoses on the patient’s health history e-file. There might be several diagnoses in the history, which are not relevant anymore or are uncertain. Therefore, if taking advantage of the e-file diagnoses list, the patient should be asked by the sleep laboratory staff on each diagnosis if the patient is aware of it and if this diagnosis is still relying to an active disease or mental or physical disorder.

## Discussion

These are to our knowledge the first recommendations for staff in sleep laboratories to manage the special CHALLENGES that arise with geriatric patients. The recommendations presented in this statement are based on a consensus of sleep physicians and sleep lab technicians from the German Sleep Society. There will still be topics which have not been touched by this group. Furthermore, the work in sleep laboratories underlies constant changes. In the future, artificial intelligence incorporated in PSG analysis programs might help to analyze electrophysical data more detailed based on the age of the patient. That is why these recommendations only represent the actual challenges and must be changed in the future according to new technical standards.

## Data Availability

Data will be made available on reasonable request. This manuscript has been written without the use of artificial intelligence.
